# Recurrence Patterns in Pilonidal Sinus Disease Surgery: Navigating Misclassifications and Early Complications

**DOI:** 10.1590/0100-6991e-20263822-en

**Published:** 2026-02-15

**Authors:** DIETRICH DOLL, CHRISTINA OETZMANN VON SACHOCZEWSKI, HENRIKE HEITMANN, THEO HACKMANN, JULIE LYNGE ANKERSEN, SUSANNE HAAS

**Affiliations:** 1 - St. Marienhospital Vechta, Procto-Surgery and Pilonidal Sinus - Vechta - Germany; 2 - University Hospital of Bonn, Surgery - Bonn - Alemanha; 3 - Randers Regional Hospital, Surgery - Randers - Dinamarca

**Keywords:** Pilonidal Sinus, Recurrence, Wound Healing, Cisto Pilonidal, Recidiva, Cicatrização de feridas

## Abstract

**Introduction:**

Wound healing problems are common after Pilonidal Sinus Disease (PSD) surgery and may come misclassified as recurrence during the first 6-12 postop. month. We hypothesized that misclassification of post-operative wound complications as recurrence would be reflected when comparing early and late annual recurrence rates.

**Methods:**

The postop. recurrence rate was analyzed in 1,254 studies encompassing 2,030 study treatment groups (n=140,472 patients), follow-up-studies <1year (n=304 studies) versus studies with longer FUP (n=950 studies). Recurrence rate was calculated for groups and subanalysis done for therapeutic strategies in this observational study.

**Results:**

First-year recurrence rate (RR) was markedly higher in the studies with <1-year follow-up compared to studies with ≥1-year follow-up (median RR 6.0%/year vs. 2.0%/year, RD=4.0%). Examining individual therapies’ recurrences after primary open treatment saw 8%/year versus 2.5%/year (RD=5.5%) RR, for primary midline closure 20%/year vs. 3.2%/year (RD=16.8%) and Pit Picking 20.7%/year vs. 4.0%/year (RD=16.7%).

**Conclusion:**

The one-year RR is containing a respectable portion of misclassifications (untrue recurrences) that may exceed the true recurrence rate by a factor of 3-5. If reliable recurrence rate needs to be determined, follow ups of <one year should be avoided. Studies of n=200 participants and larger exhibit a smaller chance of recurrence rate error and should be aimed for if possible. What does this paper add to the literature? This is the first work to quantify the amount of misclassification of “recurrences” within the first year following PSD surgery.

## RESUMO

**Introdução:**

Problemas de cicatrização de feridas são comuns após a cirurgia da Doença do Cisto Pilonidal (DCP), e podem ser mal classificados como recidiva durante os primeiros 6-12 meses pós-operatórios. Adotamos a hipótese de que a classificação errada das complicações pós-operatórias da ferida como recidivas se refletiria ao comparar as taxas anuais precoces e tardias.

**Métodos:**

A taxa de recidiva pós-operatória foi analisada em 1.254 estudos envolvendo 2.030 grupos de tratamento (n=140.472 pacientes), estudos de seguimento menor que um ano (n=304 estudos) versus estudos com seguimento mais longo (n=950 estudos). Foi calculada a taxa de recidiva para os grupos e feita a subanálise para estratégias terapêuticas neste estudo observacional.

**Resultados:**

A taxa de recidiva (TR) no primeiro ano foi significativamente mais elevada nos estudos com seguimento menor que um ano em comparação com estudos com seguimento maior que um ano (TR mediana 6,0%/ano vs. 2,0%/ano, Diferença de Risco (DR)=4,0%). A análise das recidivas das terapias individuais após o tratamento primário aberto indicou TR de 8%/ano contra 2,5%/ano (DR=5,5%), para o fechamento primário da linha média, de 20%/ano vs. 3,2%/ano (DR=16,8%) e para o Pit- Picking, de 20,7%/ano vs. 4,0%/ano (DR=16,7%).

**Conclusão:**

A TR em um ano contém uma parte relevante de classificações erradas (recorrências falsas), que podem exceder a taxa verdadeira de recorrência por um fator de 3-5. Se for necessário determinar uma taxa de recorrência confiável, devem-se evitar seguimentos de menos de um ano. Estudos com 200 participantes ou mais apresentam uma menor probabilidade de erro na taxa de recorrência e devem ser almejados. O que acrescenta este artigo à literatura? Este é o primeiro trabalho a quantificar a classificação errada de “recidivas” no primeiro ano após a cirurgia da DCP.

## INTRODUCTION

The recurrence rate of Pilonidal Sinus Disease (PSD) has been observed to follow a consistent pattern, with an approximate annual recurrence rate of 2% when all treatment methods are considered collectively^1^. This figure, however, represents a general estimate, as certain methods, such as primary open wound treatment and midline closure (the latter being generally not recommended), are associated with higher and, in some cases, unacceptable recurrence rates^2^. By contrast, advances in plastic surgical techniques, including advancement flaps (e.g., Bascom/Karydakis)^3^ and rotation flaps (e.g., Limberg/Dufourmentel), as well as other flap designs and asymmetrical closure methods, have demonstrated recurrence rates significantly below 2% annually during follow-up periods. These findings are robustly supported by evidence from randomized controlled trials (RCTs), non-RCTs, and meta-analyses conducted over recent decades^4^ ^-^ ^7^. 

Nevertheless, the literature on PSD is marked by a lack of consistency in definitions and an absence of formal agreement on what constitutes a recurrence. Prolonged wound healing following primary open treatment or wound dehiscence after closure is a common challenge, and there is ambiguity regarding the precise point at which such outcomes should be classified as failures^8^ ^,^ ^9^. The characterisation of these scenarios often involves a combination of well-defined and ambiguous criteria, as previously noted. Consequently, Allen-Mersh has recommended that recurrence should only be considered if six months have elapsed since surgery, provided that the wound had healed in between^4^. 

In the absence of this framework, applying criteria to distinguish recurrence from delayed wound healing becomes particularly challenging in the context of open wounds. In such cases, the presence of a sinus tract or hair within a porus may not be evident, and wound secretions often align with those typical of open wounds. Hair accumulation in open wounds, particularly in the lower back, is a frequent occurrence and is almost invariably found in the sacrococcygeal region. This scenario complicates the differentiation between PSD recurrence and secondary hair presence in an open wound, where the exposure of a hair nest at depth may resemble a recurrence.

Primary open pilonidal sinus wounds are observed prior to surgery in 10-15% of patients, while 5-10% of recurrences are characterised by a dehiscence exceeding 2 cm in length. Hair nests, as demonstrated by Bosche et al.^10^ ^,^ ^11^, can contain from 0 to 415 hair fragments^10^ ^-^ ^13^. However, the mere quantity of hair is not pathognomonic for distinguishing recurrence from wound dehiscence due to non-recurrence with secondary hair implantation. Emerging evidence suggests that partially open or dehiscent wounds exhibit higher recurrence rates^6^, likely due to secondary hair accumulation. This underscores the critical importance of optimising wound healing in slow or non-healing pilonidal sinus wounds^14^ ^,^ ^15^. 

The majority of wound healing complications manifest within the first six months postoperatively. Large primary open wounds, particularly when the presacral fascia is exposed and the wound is situated near the anus, often require extended periods, sometimes years, to achieve complete healing. Prolonged wound healing is a recognised factor associated with increased recurrence rates^16^. 

Given the uncertain impact of prolonged healing or surgical failure on the recurrence rate documented within the first postoperative year, this study sought to explore this relationship. We hypothesised that studies with a follow-up period of less than 12 months would report higher “recurrence rates”, as cases of prolonged healing would be misclassified as recurrences. Conversely, studies with a follow-up duration of 12 months or longer would demonstrate lower recurrence rates, as cases of prolonged healing would resolve over time. To test this hypothesis, we compared recurrence rates during the first postoperative year with those in subsequent years, calculating an annual recurrence rate by dividing the recurrence rate by the total follow-up duration.

## METHODS

The data retrieval process adhered to the Stauffer methodology^6^ and produced the results illustrated in the scatterplot diagrams.

### Search Strategy and Study Selection Criteria

To construct a comprehensive database concerning PSD, a systematic search of the existing literature employing the NCBI Medical Subject Heading (MeSH) term “pilonid*,” in addition to a combination of “dermoid” AND “cyst” was conducted. The searches were conducted across several databases, including MEDLINE, PubMed, PubMed Central, Scopus, Ovid, Embase, and the Cochrane Central Register of Controlled Trials (CENTRAL). In addition, a search using these terms in Google, Google Scholar, ResearchGate was conducted, and references cited in national and international guidelines^17^ ^-^ ^19^ were scrutinized. We also examined the references included in the bibliographies of all documents obtained through these searches. The collected documents encompassed various study types, including randomized and non-randomized trials, prospective and retrospective studies, and observational studies like cohort studies, case-control studies, cross-sectional studies, and case reports. These documents spanned the period from 1833 to 2023.

Three authors (HH, TH, DD) and one co-worker meticulously reviewed the retrieved documents to ensure compliance with inclusion criteria. These criteria necessitated the presence of information on definitive treatment, recurrence, and the duration of follow-up. The number of surgeons involved was not reported in most papers. Reports published in English, French, German, Italian, and Spanish were considered, and included publications in other languages if they provided an English abstract detailing the definitive treatment, recurrence, and follow-up time. In instances where translations were required, authors were contacted via email or ResearchGate. Exclusion criteria encompassed PSD occurrences in locations other than the presacral region, involvement of neoplastic conditions, and duplicate publication of data by the same author. Studies lacking any component of the minimal data set, which includes information on the definitive treatment strategy, recurrence, and follow-up time, were also excluded. While previous meta-analysis reports and review articles were excluded, their reference lists were scrutinized for potential additions to the evidence. Moreover, unpublished data presented in review articles were considered. Studies that inferred recurrence solely based on patients returning with recurrent disease (relying on a “return on recurrence” principle) without actively investigating most non-returners were likewise excluded.

### Data Collection, Extraction, and Quality Assessment

All identified studies underwent thorough analysis and documentation. The transcribed data were subsequently entered into a Microsoft Excel spreadsheet (Version 2019, Microsoft Corp., Redmond, WA) and subjected to verification to ensure accuracy. Each distinct therapeutic strategy reported in a study was allocated a separate line, with columns encompassing citation details, the number of included patients, therapeutic procedures, reported follow-up times, study particulars, and recurrence data. Given the variation in statistical measures used to report follow-up times across studies, we regarded mean and median reports as equivalent due to the concentration of disease incidence among young adults. For cases where minimum follow-up times were provided, these values were incorporated as reported. 

We systematically scrutinized individual studies for methodological consistency and reported results to mitigate potential bias during data synthesis. A subgroup analysis was conducted for prospective randomized controlled trials (RCTs) to ensure alignment with the broader set of studies. The recurrence rates reported in each study were then associated with the respective follow-up time, whether defined as the mean, median, center of the range, or minimum. To facilitate uniform comparison across all studies, individual patients were statistically simulated, with each study participant represented as a data sample that included recurrence status, follow-up time, and therapeutic procedure. For example, if a study included 500 patients and reported a recurrence rate of 20% for a particular therapeutic procedure, we designated 100 single samples as having recurrent disease, while the remaining 400 samples were designated as recurrence-free. Both were assigned at the follow-up as indicated above. Certain information, such as gender ratios, was excluded as it was primarily not available in detailed form in most studies. In cases where an article discussed multiple therapeutic strategies, data pertaining to each treatment strategy were separately analyzed.

### Statistical analysis

The recurrence rate per annum was computed for all studies with up to one year of follow-up, excluding those extending beyond this duration. A distinct category comprised all studies featuring a follow-up period exceeding 12 months. The recurrence rate for each study was normalized by dividing it by the total number of years under observation, thereby yielding the recurrence rate per year. In instances where the follow-up period was less than one year, the recurrence rate was divided by the number of follow-up months and then multiplied by 12. In studies with a recurrence rate exceeding the limit of 100% via this calculation method a recurrence rate of 100 was registered (i.e. 100% recurrences before end of year 1). 

For studies employing Kaplan-Meier analysis, careful attention was given to the maximum available follow-up time, and its corresponding recurrence rate was determined. Subsequently, the latter was divided by the aforementioned duration, expressed in years.

## RESULTS

An examination of a total of 1,254 studies, encompassing 2,030 treatment groups, was undertaken. A comprehensive analysis was performed, considering all treatment groups. This involved segregating the recurrence rate within the initial year (466 treatment groups) from the recurrence rates in all subsequent years (1,564 treatment groups, refer to Figure 2). The median is represented by a horizontal line in red for the one-year group and in black for the group exceeding one year.

[Fig f1]

**Figure 1 f1:**
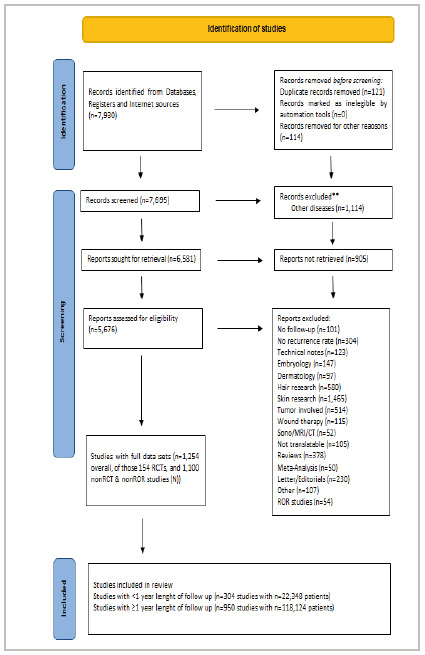
PRISMA schedule ^20^ .

**Figure 2:**
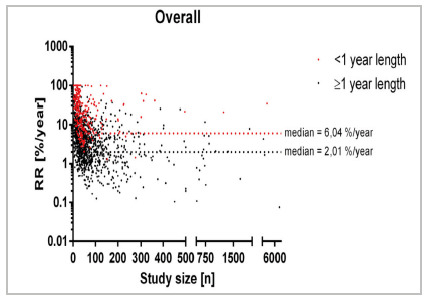
Recurrence rate overall across all therapies [%/year] in relation to study size [n] for different follow-up times. Please note that in this and the following figures a log scale is used at the y-axis leading to 622 Studies reporting a recurrence rate of 0%/year cannot be displayed, and the x-axis separates into three segments to give a better overview.

As depicted in Figure 2, the point clouds and medians are readily discernible, with the median recurrence rate at 2.01%/year and 6.04%/year, thereby denoting a threefold separation (RD=4.03%). Due to the y-axis being logarithmic, n=622 studies reporting a recurrence rate of 0%/year cannot be seen in Figure 2, as they are projected onto the X-axis for Y=log(10). They are, however, included in any further calculations.

**Figure 3:**
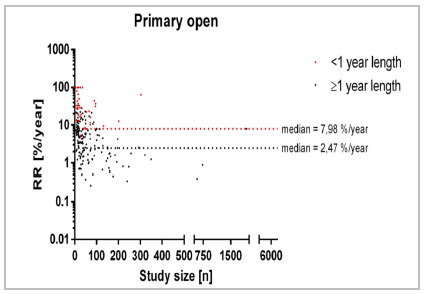
Recurrence rate across specific treatment [primary open; %/year] in relation to study size [n] for different follow-up times. 76 Studies reporting a recurrence rate of 0%/year are not displayed.

To expound further on the analysis within each treatment group, additional scrutiny was undertaken for the primary open treatment (Figure 3), encompassing 279 studies with 77 studies of <1 year of follow-up and 202 of ≥1 year follow-up. The median recurrence rate/year stood at 2.47% for follow-ups <1 year, whereas the median recurrence rate/year was 7.98% in studies with follow-up exceeding 1 year (RD=5.51%). 

**Figure 4:**
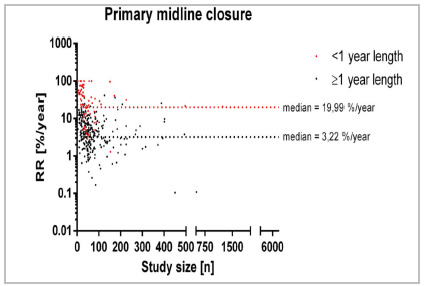
Recurrence rate across specific treatment [Primary midline closure; %/year] in relation to study size [n] for different follow-up times. 88 Studies reporting a recurrence rate of 0% are not displayed.

This effect is notably accentuated in the primary midline closure analysis encompassing 438 studies with 98 studies of <1 year follow-up and 340 of ≥1 year follow-up (Figure 4). In this context, the median recurrence rate/year stands at 19.99% in studies with <1 year follow-up whereas the recurrence rate/year was 3.22% in studies with follow-up exceeding 1 year (RD=16.77%), indicative of a heightened long-term recurrence rate as well as a conspicuously elevated events within the initial year postoperatively.

**Figure 5:**
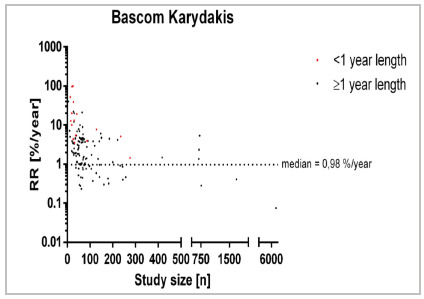
Recurrence rate across specific treatment [Bascom Karydakis; %/year] in relation to study size [n] for different follow-up times. 64 studies reporting a recurrence rate of 0%/year are not displayed.

**Figure 6:**
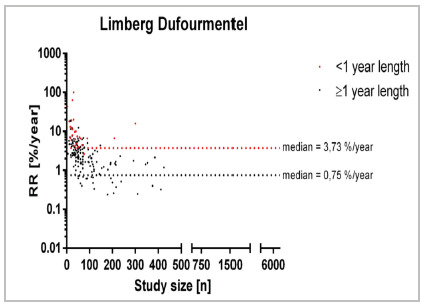
Recurrence rate across specific treatment [Limberg Dufourmentel; %/year] in relation to study size [n] for different follow-up times. 134 studies reporting a recurrence rate of 0%/year are not displayed.

The analysis of the Karydakis/ Bascoms approach encompassed 185 studies with 34 studies of <1 year follow-up and 151 of ≥1 year follow-up (Figure 5). The median recurrence rate/year for studies of < one year follow-up was located at 0% whereas it was 0.98% in studies with follow-up exceeding 1 year (RD=0.98%). Figure 5 A parallel observation emerges within the Limberg / Dufourmentel cohort encompassing 313 studies with 57 studies of <1 year follow-up and 256 of ≥1 year follow-up (Figure 6). The median recurrence rate/year for studies of < one year follow-up was 3.73% whereas it was 0.75% in studies with follow-up exceeding 1 year (RD=2.98%).

The analysis of marsupialization encompassed 100 studies with 25 studies of <1 year follow-up and 75 of ≥1 year follow-up, demonstrated a 0% median recurrence rate/year in the <1 year follow-up studies compared to prolonged median recurrence rate/year of 1.2% in studies with follow-up exceeding one year (RD=1.19%). 

**Figure 7:**
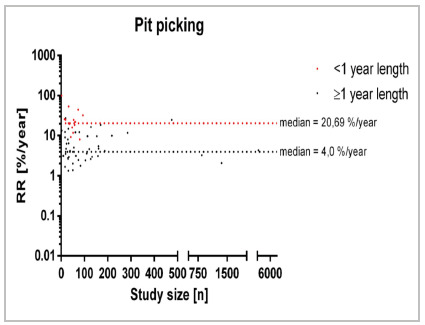
Recurrence rate across specific treatment [Pit picking; %/year] in relation to study size [n] for different follow-up times. 7 studies reporting a recurrence rate of 0%/year are not displayed.

Lastly, the analysis of Pit Picking (Figure 7) encompassed 68 studies with 17 studies of <1 year follow-up and 51 studies of ≥1 year follow-up. Here, a median recurrence rate/year was 20.69% in studies with <1 year follow-up compared to a median recurrence rate/year of 4.0% in the studies with a follow-up exceeding one year (RD=16.69%). Upon scrutinizing the dispersion around the provided medians, minimal dispersion is observed, notably beneath a study size of n=100 patients. Beyond a study size ranging from 200 to 300 patients, the recurrence rate per annum results appear to converge closer to the median. Beyond a study size of n=200 patients, no recurrence rate per annum surpasses 100.

## DISCUSSION

An extensive review of 1,254 studies comprising 2,030 treatment groups and involving 140,472 patients treated over 180 years unveils several intriguing findings. This dataset provides an unparalleled opportunity to examine recurrence rate patterns both across treatment groups and within specific surgical approaches.

For the first time, we identify that recurrence (and/or wound-related complications addressed as recurrence) are disproportionately elevated during the months leading up to the first postoperative year. This observation is consistent across the entire dataset and within individual treatment subgroups, revealing a two- to tenfold increase in reported recurrences within this timeframe. This finding emphasises that the first postoperative year represents a distinctly vulnerable period. 

Several hypotheses may account for this pronounced early recurrence pattern: 

The elevated recurrence rates observed in the first postoperative year can be attributed to several potential factors. Initially, issues such as unstable scars, impaired healing, or early trauma may mimic the clinical presentation of true recurrences, complicating their differentiation. In addition, genuine recurrences may indeed occur, but it remains uncertain whether the determinants of recurrence during the first year are comparable to those in subsequent years. True recurrences originating from within the wound often manifest earlier, though they may become less apparent over time, as their clinical onset tends to align with the initial postoperative period.

In contrast, recurrence resulting from the external reinsertion of axial hair fragments represents a distinctly different pathological mechanism. While axial hair force remains relatively constant during the first postoperative year^11^ ^,^ ^13^, the gradual maturation of scar tissue enhances wound strength and alters the dynamic between the skin’s resilience and external factors. Over time, these biological processes are thought to contribute to a reduction in vulnerability, though the interplay between wound and skin properties in this context remains an area requiring further investigation. Wound rupture tends to occur more frequently in the early postoperative period, primarily due to the minimal tensile strength of scars at the onset of the healing process. Initially, fibrin deposition provides a provisional scaffold, followed by collagen production driven by fibroblast activity. As healing progresses, type III collagen undergoes maturation into the more robust type I collagen, characterized by stronger interlinking cross-bonds. By approximately 42 days postoperatively, scar tissue achieves about 70% of its tensile strength, as evidenced by studies in animal models^22^ ^,^ ^23^. Further maturation enhances this strength to around 80-90% by three months, but beyond this plateau, full preoperative tensile strength is not regained, as supported by available data^22^ ^,^ ^24^. 

Given these dynamics, wound healing complications are particularly prevalent within the first three months after surgery, coinciding with an increased risk of recurrence during this period. In contrast, recurrences that manifest later may be driven by different mechanisms. While environmental factors such as wet surfaces and sweating are not believed to facilitate the injection of short hair fragments into the skin^25^ ^,^ ^26^, the resilience of scar tissue-whether early or more mature-against such penetration remains largely unexplored. This critical question will be the focus of a planned study aimed at elucidating the interplay between scar maturation and external mechanical forces.

Four distinct mechanisms may contribute to recurrence-like situations, each warranting detailed consideration: 

Surgical dehiscence may present as postoperative wound reopening in a previously closed surgical site, resulting from complications such as hematoma, seroma, infection, ischemic non-healing, or excessive wound tension. Once the wound reopens, hair may embed secondarily, mimicking the appearance of recurrence. This can complicate clinical differentiation, as the wound may subsequently be misinterpreted as a recurrence caused by hair infiltration.

True recurrence from within the wound can manifest postoperatively as wound dehiscence, often originating from infections linked to residual sinus tracts or unsterilized hair left in the surgical field. The likelihood of leaving residual tracts can be minimized through three key interventions: ensuring complete drainage of acute pilonidal sinus disease (PSD) to allow resolution of infection and tissue edema^27^, operating only under conditions of reduced infection, and using tools such as methylene blue or other staining agents to precisely delineate sinus tracts intraoperatively^28^. Interestingly, PSD surgery performed during elective daytime conditions has been associated with a lower rate of recurrence, though the reasons for this remain unclear^29^. 

If a wound closes or heals over embedded hair, it may later rupture, resulting in partial or complete reopening or the development of fistulas of varying sizes. Such events are predominantly observed within the scar area of the original wound. If pre-existing wound healing issues are present, they may predispose the wound to secondary hair embedding, creating a cycle of recurrence-like phenomena. Clinically, it may be impossible to distinguish whether hair is entering or exiting the wound.

Even after complete wound healing, traumatic wound dehiscence may occur due to the inability of a still-maturing scar to withstand external mechanical forces. This type of rupture, resulting from external trauma, impairs an otherwise healthy wound. For instance, in the gluteal region, where hair accumulation is common, open wounds can inadvertently trap hair, leading to later recurrence. Open or partially open wounds are especially prone to recurrence^16^, underscoring the intricate interplay between wound rupture, healing complications, and recurrence.

In some cases, a new disease process-referred to as repeat Pilonidal Sinus Disease-may occur due to the injection of hair into previously healed scar tissue, the neo-Rima, or adjacent skin areas. This leads to the formation of a new tract, distinguishable from the original disease process. Hair may be identified within the newly formed tract, and while personal risk factors such as axial hair force or familial predisposition remain constant, this represents a new pathological entity rather than a recurrence of the original disease. However, whether this condition should be classified as a recurrence or a distinct disease remains an unresolved question.

The situations described above are challenging to differentiate, as recurrences originating beneath the skin may either present within the old scar or emerge far from the midline, while new pilonidal sinus disease (PSD) can manifest as small tracts located away from the original scar. Surgical wound rupture and traumatic wound rupture, which typically occur within a short period following the procedure, likely contribute to the disproportionately high recurrence rates observed in the first postoperative year across all surgical methods.

However, these factors alone may not fully explain the phenomenon, and other unknown influences cannot be excluded. Notably, there is limited understanding of critical factors such as skin resistance to micropenetration and the mechanical resilience of human scar tissue-both of which remain subjects of ongoing research. These gaps in knowledge highlight the complexity of wound healing and recurrence patterns.

Several factors likely interact to produce the observed effects, but one particularly striking observation stands out. Across 1,240 publications authored by over 3,000 surgeons, these recurrence-like phenomena were overwhelmingly categorized as recurrences and interpreted as therapeutic failures. This reflects an extraordinary level of professional transparency and self-criticism. By openly acknowledging these adverse outcomes, often erring on the side of caution to critically evaluate their own results, these surgeons demonstrated a remarkable commitment to improving patient care. This honesty not only fosters trust but also provides a unique opportunity to analyze and understand the underlying dynamics reflected in the data.

### Limitations

The limitations of this analysis stem primarily from its retrospective nature, as the studies included were not prospectively planned. These inherent constraints have been thoroughly discussed in prior evaluations of this comprehensive Pilonidal Sinus Disease dataset^6^. Despite these challenges, the fundamental parameters of surgical methods and study sizes were clearly defined in nearly all studies, ensuring a solid foundation for analysis.

One area of difficulty lies in accurately determining recurrence rates, particularly in the early postoperative months. During this period, distinguishing between surgical complications and genuine early recurrence is nearly impossible, introducing a degree of observation bias. Nonetheless, this very parameter was a focal point of the investigation and is central to the study’s conclusions.

Return-on-recurrence (ROR) scenarios, which systematically underestimate recurrence rates, have been examined in previous research^30^. To ensure accuracy, studies suspected of employing ROR methods were excluded from this analysis. While randomized controlled trials (RCTs) are often regarded as the gold standard of research, their contribution to this dataset is limited. RCTs represented only 154 studies with 15,283 patients, compared to 1,100 non-RCT studies involving 125,189 patients. Additionally, RCTs are not without their challenges, with fragile statistical significance reported in over 53% of cases^31^, and instances of false^32^ or even falsified^33^ data further complicating their reliability. As a result, the influence of RCTs on the overall findings of this study is minimal.

Based on the available data, recurrence in clinical trials is most reliably defined as disease re-emerging after complete wound healing, typically more than 12 months following curative surgery. This aligns with common sense and standard practice^4^ ^,^ ^34^, making Kaplan-Meier recurrence rates beyond the first year, particularly those measured at five- and ten-year intervals, the most dependable metrics for assessing long-term outcomes^35^. 

## CONCLUSION

It is unmistakably clear that for an accurate portrayal of the true recurrence rate in Pilonidal Sinus Disease, follow-up must extend well beyond the first year, and ideally, encompass several years. Without this, we risk underestimating or misrepresenting the actual recurrence patterns. Crucially, precise and universally accepted definitions of what constitutes recurrence must be established and rigorously applied in all future studies to prevent inconsistencies and misinterpretations.

Assessing recurrence rates too early invites significant error, as the first postoperative year appears to be a critical yet confounding period. During this time, unidentified factors-or perhaps an interplay of multiple mechanisms-exacerbate recurrence rates or create situations easily mistaken for true recurrences. The result is an amplified recurrence rate that obscures the genuine long-term trends. This phenomenon demands urgent and focused investigation to identify and address these early postoperative variables.

Moreover, evidence strongly suggests that studies with larger cohorts (n=200 or more patients) yield more reliable and precise recurrence data. Smaller sample sizes increase the margin of error, undermining the robustness of findings and making this an essential consideration in the design of future research.

The strikingly elevated recurrence rate within the first year is a glaring anomaly that cannot be ignored. Its origins-whether mechanical, biological, or methodological-remain elusive and represent a critical frontier for future inquiry. Until these uncertainties are resolved, the recurrence rates reported in early postoperative studies should be viewed with caution, and the medical community must strive for long-term, high-quality data to guide clinical decisions and improve patient outcomes. The stakes are too high for anything less.
